# Congener Profiles and Source-Wise Phase Partitioning Analysis of PCDDs/Fs and PCBs in Gyeonggi-Do Ambient Air, South Korea

**DOI:** 10.3390/ijerph111111065

**Published:** 2014-10-24

**Authors:** Jongwon Heo, Donggi Kim, Gangwoong Lee

**Affiliations:** 1 Gyeonggi-Do Institute of Health and Environment, Suwon 440-290, Korea; E-Mails: heo7777@gg.go.kr (J.H.); kimdg@gg.go.kr (D.K.); 2 Department of Environmental Science, Hankuk University of Foreign Studies, Yongin 449-791, Korea

**Keywords:** PCDDs/Fs, PCBs, congener profiles, gas-particle partitioning, Korea

## Abstract

The atmospheric concentrations and gas–particle partitioning of polychlorinated dibenzo-p-dioxins and furans (PCDDs/Fs) and polychlorinated biphenyls (PCBs) were investigated at two sites (Suwon and Ansan) in Gyeonggi-do, a heavily industrialized area of Korea, during the year 2010. The sum level (Σ17) of PCDDs/Fs and dioxin-like PCBs (dl-PCBs) in the ambient air at Suwon and Ansan ranged from 0.04 to 0.30 pg-TEQ·m^−3^ (geometric mean: 0.09 pg-TEQ·m^−3^) and 0.17 to 0.63 pg-TEQ·m^−3^ (geometric mean: 0.36 pg-TEQ·m^−3^), respectively. Moreover, the geometric mean concentrations of Σ180 PCBs at Suwon and Ansan were 233.6 pg·m^−3^ and 274.2 pg·m^−3^, respectively, and di-chlorinated biphenyls and tri-chlorinated biphenyls were the predominant homologs. Among the PCB congeners, 3,3'-dichlorobiphenyl (PCB-11) was the dominant species at both sites during all sampling periods, comprising up to 15.1% of Σ180 PCBs at Ansan and 24.6% at Suwon. We evaluated their gas-to-particle equilibriums by conducting regression between the particle–gas partition coefficient K_p_ (m^3^·ug^−1^) and the corresponding subcooled liquid vapor pressure (P_L_°). The slope (m) values for log–log plots of K_p_ *vs.* P_L_° were steeper in industrial areas owing to local source proximity. Moreover, owing to enhanced emissions from combustion-related sources at low temperatures, PCDD/Fs exhibited the largest deviation from the regression line of the particle–gas partition coefficient. Incinerators were found to be the primary emission source of atmospheric PCDDs/Fs, whereas re-evaporation from pre-existing environmental loads (e.g., storage areas or spilled soil and water bodies) was the dominant source for PCBs.

## 1. Introduction

Polychlorinated biphenyls (PCBs) were first synthesized by Schmidt and Shultz [1], and have been used widely in industrial applications since the 1920s owing to their chemical stability, including their high heat resistance and nonflammability [2,3]. For example, they have been used in electrical equipment, as dielectric fluids in capacitors and transformers, over the past 50 years. Polychlorinated dibenzo-p-dioxins and furans (PCDDs/Fs) were first detected in incineration ash [4]. PCDDs/Fs are highly toxic and persistent when released into the environment through combustion processes associated with the production of chlorinated chemicals; accordingly, incinerators represent an important source of PCDD/Fs pollution in Korea [5,6]. Because PCBs and PCDDs/Fs are persistent organic pollutants (POPs) with significant bioaccumulation in environmental systems and long-range transport [7], many countries are greatly concerned about these compounds. PCBs are still produced and used in Korea, whereas PCDD/Fs are almost always inadvertent byproducts of combustion or chemical production. The Stockholm Convention on POPs aims to ban the production and use of PCBs worldwide and enforce the environmental monitoring of PCBs to protect both human health and the environment. Despite this prohibition and concern, PCBs and PCDDs/Fs continue to be detected in the ambient air, and their concentrations in the environment are not negligible. In particular, PCBs are being released into the atmospheric environment via volatilization from uncontrolled landfill and illegal dumping of wastes or parts containing PCBs and via emissions from stored and incomplete incineration of certain wastes [8,9,10]. Thus, sites within urban and commercial areas may be influenced by localized PCB sources [2,7].

Atmospheric transport is an important pathway for delivery of POPs to water and terrestrial surfaces, and the gas-particle partitioning process between the vapor and solid phases for individual congeners is a very important factor in determining the fate of POPs in the atmosphere [11,12]. To understand POP gas-particle partitioning, many researchers have suggested an adsorption-absorption model that depends on particle surface properties and adopts the octanol-air partitioning coefficient (K_OA_) as an alternative to the subcooled liquid vapor pressure (P_L_°) [10,13]. However, few data are available regarding the atmospheric gas–particle concentration of individual PCBs and PCDDs/Fs simultaneously or the temporal distribution of PCBs in Korea [3]. Accordingly, most PCB studies have focused on dioxin-like PCBs identified by the World Health Organization (WHO) and on seven congeners that are found commonly in the environment and are considered to be indicators of the degree of contamination.

In the present study, we investigated the individual PCB 180 and PCDD/F 17 congeners and gas/particle concentration to estimate partition of gas and particles and their characteristic behaviors in urban and industrial areas in Gyeonggi-do, Korea, where their total (gas and particle) concentrations were compared in passive and active samplers to a previously published work [14].

## 2. Material and Methods

The sampling sites and sampling and analysis methods are identical to those of a previous study using active high-volume air samplers; accordingly, selected sampling parameters and analysis methods have been published previously [14]. The sampled air volume per week was in the range 940–1423 m^3^. Samples at Suwon (SW) and Ansan (AS) were collected for 380 days and 173 days, respectively, starting in February, 2010. Table 1 lists the sampling periods at the sampling sites.

**Table 1:** Sampling periods at the sampling sites.

**Sampling Event**	**Suwon**																												
	**SW1**	**SW2**		**SW3**	**SW4**		**SW5**	**SW6**		**SW7**	**SW8**		**SW9**	**SW10**		**SW11**		**SW12**	**SW13**		**SW14**		**SW15**	**SW16**		**SW17**	**SW18**		**SW19**
Date (Year: 2010)	2.03	2.24		3.18	3.31		4.07	4.23		5.07	5.14		5.28	6.11		6.28		7.12	7.26		8.16		9.06	9.27		10.13	11.01		11.19
	–	–		–	–		–	–		–	–		–	–		–		–	–		–		–	–		–	–		–
	2.24	3.18		3.31	4.07		4.23	5.07		5.14	5.28		6.11	6.28		7.12		7.26	8.16		9.06		9.27	10.13		11.01	11.19		12.08
**Sampling Event**	**Ansan**																												
	**AS1**		**AS2**			**AS3**			**AS4**			**AS5**			**AS6**		**AS7**			**AS8**		**AS9**			**AS10**			**AS11**	
Date (Year: 2010)	2.24		3.18			3.31			4.16			4.30			5.14		5.28			6.11		6.28			7.12			7.26	
	–		–			–			–			–			–		–			–		–			–			–	
	3.18		3.31			4.16			4.30			5.14			5.28		6.11			6.28		7.12			7.26			8.16	

The PCDDs/Fs and 180 individual congeners of PCBs were analyzed according to U.S. EPA 1613 and 1668 B, respectively, using an HRGC/HRMS (Agilent 6890 and Autospec NT; Micromass, UK) with sp2331 (60 m × 0.32 mm × 0.2 μm; Sigma–Aldrich, Supelco; Bellefonte, PA, USA) column for PCDDs/Fs and DB-5MS (60 m × 0.2 mm × 0.25 μm; J and W Scientific, Santa Clara, CA, USA) for PCBs.

Laboratory blanks and field blanks were analyzed for every sampling event, and highly chlorinated congeners were detected primarily above the limit of detection (LOD). Blank values were subtracted only in the case of sampled concentrations higher than the LOD. The LOD was defined as three times the standard deviation of the lowest calibration points, which were 0.04–0.46 (average: 0.15) pg/sample for PCDDs and 0.00–0.29 pg/sample (average: 0.14) for PCB. The average recoveries of the surrogate PCDDs/Fs and PCBs were in the range 80.3 ± 11.0 to 117.4% ± 25.0% and 76.8 ± 20.6 to 119.1% ± 12.6%, respectively; these values meet the sample recovery requirements of EPA 1613 and EPA 1668 B.

## 3. Results and Discussion

### 3.1. Concentration of PCDDs/Fs and PCBs in the Ambient Air

**Figure 1:**
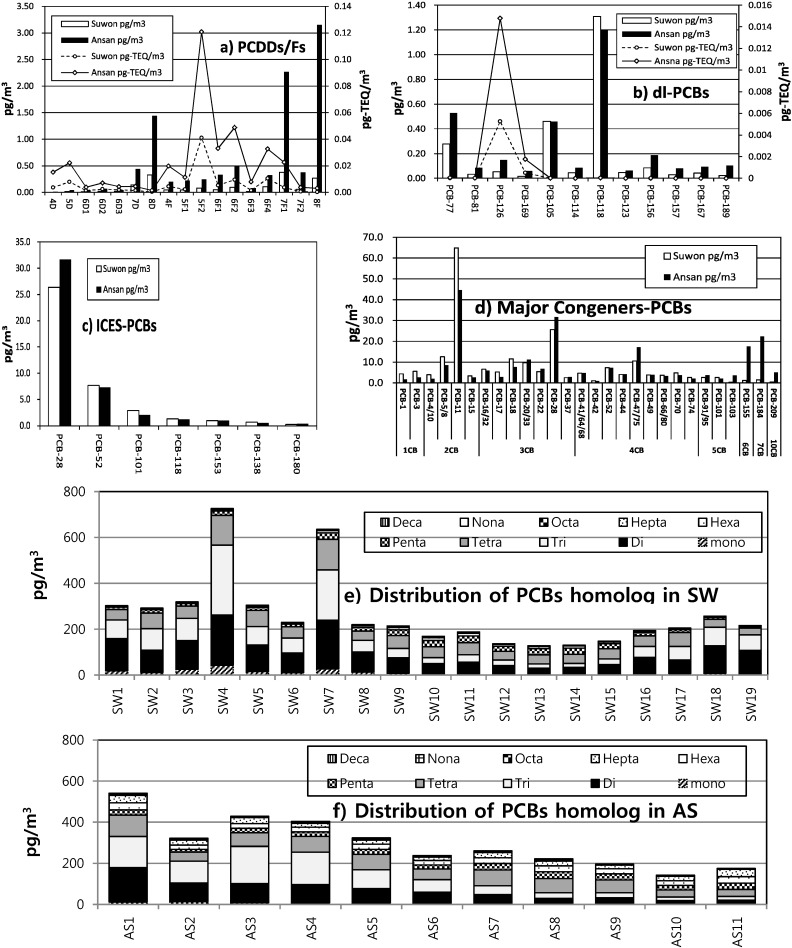
Average concentrations of PCDDs/Fs, dl-PCBs, and ICES-PCB congeners and PCB homolog groups at the Suwon and Ansan locations (4D: 2,3,7,8-TCDD, 5D: 1,2,3,7,8-PCDD, 6D1: 1,2,3,4,7,8-HxCDD, 6D2: 1,2,3,6,7,8-HxCDD, 6D3: 1,2,3,7,8,9-HxCDD, 7D: 1,2,3,4,6,7,8-HpCDD, 8D: OCDD, 4F: 2,3,7,8-TCDF, 5F1: 1,2,3,7,8-PCDF, 5F2: 2,3,4,7,8-PCDF, 6F1: 1,2,3,4,7,8-HxCDF, 6F2: 1,2,3,6,7,8-HxCDF, 6F3: 2,3,4,6,7, 8-HxCDF, 6F4: 1,2,3,7,8,9-HxCDF, 7F1: 1,2,3,4,6,7,8-HpCDF, 7F2: 1,2,3,4,7,8, 9-HpCDFs, 8F: OCDF).

The notation ΣPCDDs/Fs and ΣPCBs refers to the sum of the 2,3,7,8 substituted PCDDs/Fs for 17 species and 180 congeners of PCBs. The International Council for the Exploration of the Seas (ICES-PCB) designated the 7 PCB (28, 52, 101, 118, 138, 153, 180) congener group commonly found in the environment as an indicator of the degree of PCB contamination. The toxic equivalent quantity (TEQ) value for PCDDs/Fs obtained from the International Toxic Equivalent Factors (I-TEF) and TEQ for dl-PCBs used the 2005 World Health Organization TEF. The mean concentration TEQs of PCDDs/Fs and ΣPCBs for SW and AS air are given in Table 2 and Figure 1a.

**Table 2:** PCB and PCDDs/Fs concentrations in Asia since the year 2000.

***PCBs***					
**City**	**Country/Area (Sampling date; Reference)**	**No. ^b^**	**No. ^c^**	**Mean ^d^ pg/m^3^**	
**Suwon**	Korea/Urban (2010; this study)	19	180	Σ **PCB** = 233.60	Gas = 221.41
					Particle = 6.10
		19	7	Σ**ICES-PCB** = 34.08	Gas = 32.06
					Particle = 0.63
		19	12	Σ**dl-PCB** = 2.28 (0.006 pg-TEQ/m^3^)	Gas = 2.00 (0.004 pg-TEQ/m^3^)
					Particle = 0.15 (0.001 pg-TEQ/m^3^)
**Ansan**	Korea/Industrial (2010; this study)	11	180	Σ**PCB** = 274.15	Gas = 251.77
					Particle = 12.37
		11	7	Σ **ICES-PCB** = 36.45	Gas = 33.70
					Particle = 0.77
		11	12	Σ**dl-PCB** = 4.24 (0.023 pg-TEQ/m^3^)	Gas = 3.66 (0.016 pg-TEQ/m^3^)
					Particle = 0.35 (0.004 pg-TEQ/m^3^)
**Seoul**	Korea/Urban (1999–2000; Yeo *et al.* 2004) [15]	14	41	Σ**PCB** = 130.41	
**Ansung**	Korea/Rural (1999–2000; Yeo *et al.* 2004) [15]	14	41	Σ**PCB** = 39.65	
**30 Cities ^a^**	Korea/30 Areas (2008; Hogarh *et al.* 2011) [16]	30	202	Σ**PCB** = 155.77	
**58 Cities ^a^**	Japan/58 Areas (2008; Hogarh *et al.* 2011) [16]	58	202	Σ**PCB** = 315.02	
**Nationwide**	Japan/102 Areas (2002; MOE, Japan. 2006) [17]	34	-	Σ**PCB** = 100.00	
**Nationwide**	Japan/69 Areas (2003; MOE, Japan. 2006) [17]	69	-	Σ**PCB** = 260.00	
**Nationwide**	Japan/74 Areas (2004; MOE, Japan. 2006) [17]	74	-	Σ**PCB** = 240.00	
**20 Cities**	China/20 Areas (2008; Hogarh *et al.* 2011 ^a^) [16]	20	202	Σ**PCB** = 1,512.58	
**Taichung**	Taiwan/Subrural (2008;Hogarh *et al.* 2011 ^a^) [16]	1	202	Σ**PCB** = 316.51	
***PCDDs/Fs***					
**City**	**Country/Area (Sampling date; Reference)**	**No. ^b^**	**No. ^c^**	**Mean ^d^ I TEQ pg/m^3^**	
**Suwon**	Korea/Urban(2010; this study)	19	17	ΣPCDDs/Fs = 0.086	Σ PCDDs/Fs (gas) = 0.022
					Σ PCDDs/Fs (particle) = 0.055
**Ansan**	Korea/Industrial (2010; this study)	11	17	ΣPCDDs/Fs = 0.357	Σ PCDDs/Fs (gas) = 0.109
					Σ PCDDs/Fs (particle) = 0.201
**Nationwide**	Japan/48 Areas (2002; MOE, Japan. 2006) [17]	48	17	ΣPCDDs/Fs = 0.160	
**Nationwide**	Japan/48 Areas (2003; MOE, Japan. 2006) [17]	48	17	ΣPCDDs/Fs = 0.077	
**Nationwide**	Japan/48 Areas (2004; MOE, Japan. 2006) [17]	48	17	ΣPCDDs/Fs = 0.074	
**Shanghai**	China/Urban-traffic center (2006; Li *et al.* 2008) [18]	7	17	ΣPCDDs/Fs = 0.289	
**Shanghiai**	China/Industrial (2006; Li *et al.* 2008) [18]	7	17	ΣPCDDs/Fs = 0.497	
**Beijing ^e^**	China/Urban (2006; Li *et al.* 2008) [19]	12	17	ΣPCDDs/Fs = 0.268	
**Hangzohou**	China/Agriculture-Urban (2007–2008; Xu *et al.* 2009) [20]	24	17	ΣPCDDs/Fs = 0.295	
**Guangzhou**	China/Industrial (2004; Yu *et al.* 2006) [21]	6	17	ΣPCDDs/Fs = 0.769	
**Kaohsiung**	Taiwan/Industrial (2005; Wang *et al.* 2009) [22]	40	17	ΣPCDDs/Fs = 0.074	

Notes: **^a^** Data obtained by passive air sampler; **^b^** Number of samples; **^c^** Number of congener analysis; **^d^** Geometric mean (GM); **^e^** Arithmetic mean (AM).

The TEQ levels of PCDDs/Fs in developing areas in Asia were higher than those in many cities in Europe and America [23,24,25,26]. The concentrations of ΣPCDDs/Fs based on the TEQ in the ambient air of SW and AS ranged from 0.033 to 0.284 pg-TEQ·m^−3^ (Arithmetic mean (AM): 0.098, Geometric mean (GM): 0.086 pg-TEQ·m^−3^) and from 0.153 to 0.613 pg-TEQ·m^−3^ (AM: 0.379, GM: 0.357 pg-TEQ·m^−3^), respectively. TEQs in AS are higher than those in SW, confirming the existence of sources of PCDDs/Fs in the industrial area.

These concentrations of PCDDs/Fs in the Ansan ambient air are similar to those in other industrialized Asian countries [18,19] but higher than those of Japan [17]. Mean PCDD/F values (0.144 pg-TEQ·m^−3^) are in agreement with the ranges found in a previous study [27] at urban/industrial sites, in the range ~10–100 pg·m^−3^ (ΣTEQ ~100–400 fg·m^−3^).

In East Asia (Table 2), the average atmospheric PCB concentrations in Japan (315 pg·m^−3^) and Korea (156 pg·m^−3^) were found to be comparable, whereas that of China (1513 pg·m^−3^) was much higher [16,28]. The concentration of ΣPCBs in the ambient air at SW and AS ranged from 127.6 to 725.9 pg·m^−3^ (AM: 263.9, GM: 233.6 pg·m^−3^) and from 142.5 to 540.7 pg·m^−3 ^ (AM: 295.7, GM: 274.15 pg·m^−3^), respectively. Regardless of whether in urban and industrial areas, the average ΣPCB and ICES PCB concentrations were found to be at similar levels; this has been postulated to have been caused primarily by widespread emissions from areas in which associated compounds have been used, stored, spilled, or atmospherically deposited [29,30].

However, the mean concentration of dl-PCBs at SW and AS were determined to be 2.28 and 4.24 pg·m^−3^ (0.006 and 0.023 pg-TEQ·m^−3^), respectively. We conducted one-way ANOVA (analysis of variance) of concentration for PCDDs/Fs and dl-PCBs for the same sampling period. The results for PCDDs/Fs and dl-PCBs produced ρ = 3.3 × 10^−6^ and ρ = 9.3 × 10^−5^, respectively, and these values exhibit a significant site difference at the 95% confidence level. However, in the case of the ICES-PCBs (ρ = 0.70) and total PCBs (ρ = 0.81), no significant site difference was apparent in the ANOVA results. This indicates that the emissions of dl-PCBs into the atmosphere likely occurred primarily in the industrialized region (AS), because the major potential of sources of dl-PCBs and PCDDs/Fs are emissions from manufacturing plants and waste incinerators [23,31].

### 3.2. Profile Congeners of PCDDs/Fs and PCBs and in the Ambient Air

**PCDDs/Fs:** The main contributors to the Σ2,3,7,8-PCDDs/Fs were OCDDs (SW: 19.6%, AS: 14.8%), OCDFs (SW: 15.7%, AS: 32.2%), and 1,2,3,4,6,7,8-HpCDF (SW: 22.5%, AS: 23.2%). SW presents a typical pattern for urban areas, with OCDD being the most prevalent congener [27,32], whereas OCDF was shown to be the largest contributor in the AS industrial area. In previous studies [33], OCDF was found primarily in incinerator neighborhoods and industrial complex areas.

With respect to the I-TEQ, the congener 2,3,4,7,8-PeCDF was the dominant contributor to ΣITEQs, accounting for approximately 30%–45%. The congener 2,3,4,7,8-PeCDF was reported to be the dominant I-TEQ contributor in most primary and secondary PCDD/F emission sources [20,34], and could be considered the single most important contributor according to most reports [27].

**PCBs:** Among the large number of PCBs analyzed, the compositions of dl-PCBs, ICES-PCBs, and major PCBs that contributed more than 1% of ΣPCBs are reported in Figure 1. The dl-PCBs were similar to those of previous studies, illustrated in Figure 1b [35,36], with higher contributions of congeners PCB-118, PCB-105, and PCB-77. PCB-126 was found to be the main contributor to the average WHO-TEQ value owing to its high TEF, and was the most abundant congener in the ambient air [37].

The ICES-PCB congener profile in Figure 1c shows that concentration of ICES-PCBs decrease with increased chlorine number for all samples. The percentage of ICES-PCBs to ΣPCBs was about 15%, and the coefficient of determination (R^2^) between ΣPCBs and ICES-PCBs was 0.92 (*p* < 0.001), which indicate that total PCB concentration can be deduced from ICES-PCB measurements.

The major PCB congeners exceeding 1% of ΣPCBs are shown in Figure 1d. In both areas, PCB-11, 28/31, 47/45, 5/8, 20/33, 18, 52, and 22, in order from high to low levels, were found to account for about 50% of ΣPCBs. A relatively high concentration of PCB-11 was found in the atmosphere, accounting for 15.1% and 24.6% of the ΣPCBs at AS and SW, respectively. The percentage of ΣPCBs made up by the PCB-11 congener was higher than that made up by the sum of seven indicator congeners (ICES-PCBs), and the correlation coefficients (R^2^) between PCB-11 and total PCBs taken pairwise were found to be 0.72 for SW and 0.91 for AS, with high significance (*p* < 0.001); therefore, even PCB-11 could be regarded as an indicator that can quantify total PCBs. PCB-11 concentrations were found to be generally higher at SW, whereas many of the other congeners are typically more abundant at AS. PCB-11 often exists at high concentrations, and was recently reported in the ambient air of various regions [37,38,39,40]. This PCB-11 may originate primarily from the yellow dye used in printing and painting applications, and from the degradation of higher chlorinated PCB congeners [40,41].

The compositional homolog profiles for the PCBs of each sample are shown in Figure 1e–f. The 2-CBs, 3-CBs, and 4-CBs were the dominant homolog groups, accounting for 10.4%–49.7% (mean: 28.7%), 10.56%–42.24% (mean: 24.5%), and 13.3%–31.6% (mean: 22.9%) of ΣPCBs, respectively. The PCBs in both cities are enriched in the lower chlorinated PCB congeners. This homolog pattern is consistent with the PCB composition reported in a previous study [37]. Owing to the low partitioning characteristics (octanol-air coefficient; K_OA_) and high vapor pressure, low molecular PCBs have been shown to exhibit higher concentrations than high molecular PCBs [3,42] in the atmosphere at both AS and SW.

The homologs of 6CB–10CB were found to contribute more extensively at AS than at SW. Moreover, among the major congeners, PCB-155 (HexaCB), 184 (HeptaCB), 209 (DecaCB) were found to be enriched at AS relative to SW. These results indicate that high molecular weight (HMW) PCBs are likely to remain in the vicinity of local sources, whereas low molecular weight (LMW) PCBs are more likely to be transported over long ranges [43].

### 3.3. Gas/Particle Partitioning of PCDDs/Fs and dl-PCBs in the Atmosphere

PCDDs/Fs were typically associated with the particle phase, whereas most of the PCBs were gas-phase chemicals with relatively high vapor pressure. Commonly, the higher the molecular weight of a chemical with increasing number of chlorine, the higher the octanol-partitioning coefficient (K_OW_) and the more easily the chemical is related to the particle phase. To avoid breakthrough of the PUF-plugs and allow estimation of the gas partitioning of the semi-volatile organic compounds (SOCs), we focus on the PCDDs/Fs and dl-PCBs that exhibit low concentrations in ambient air. PCDDs/Fs and dl-PCBs are typically present at low concentrations compared to other SVOCs. Lee *et al*. (1999) [44] stated that no breakthrough occurred at volumes of 1000 m^3^ for weekly and mean weekly temperatures of >15 °C, even for LMW-PCDDs/Fs.

Partitioning of SOCs between the gas and particle phases affects their rates of wet and dry deposition and their long-range transport [45,46]. To evaluate these two phase partitions, the particle-gas partition coefficient, K_p_ (m^3^·μg^−1^) [13,47], and the regression of K_p_ *versus* the corresponding subcooled liquid vapor pressure (P_L_°) were used in previous studies:

Log K_p_ = log [(F/TSP)/A] = m·log P_L_° + b (1)

where F is the particle-phase concentration (pg·m^−3^), A is the gas-phase concentration (pg·m^−3^), and TSP is the concentration of total suspended particle matter (μg·m^−3^). The slope (m) and intercept (b) calculated from the log K_p_ *vs.* log P_L_° plots are useful regression parameters that can describe the equilibrium state of adsorption/absorption.

At equilibrium state, the slope (m) value for adsorption or absorption should be close to −1 [13]. Some studies have indicated that such a slope in the regression of thr log K_p_ *vs.* log P_L_° is not necessary to describe equilibrium partitioning [30,48]. Here, P_L_° values for PCDDs/Fs and PCBs were determined by the GC-retention time index [49] and data from a previous study [50], respectively. These values were modified at average air temperature during the sampling period.

**Figure 2:**
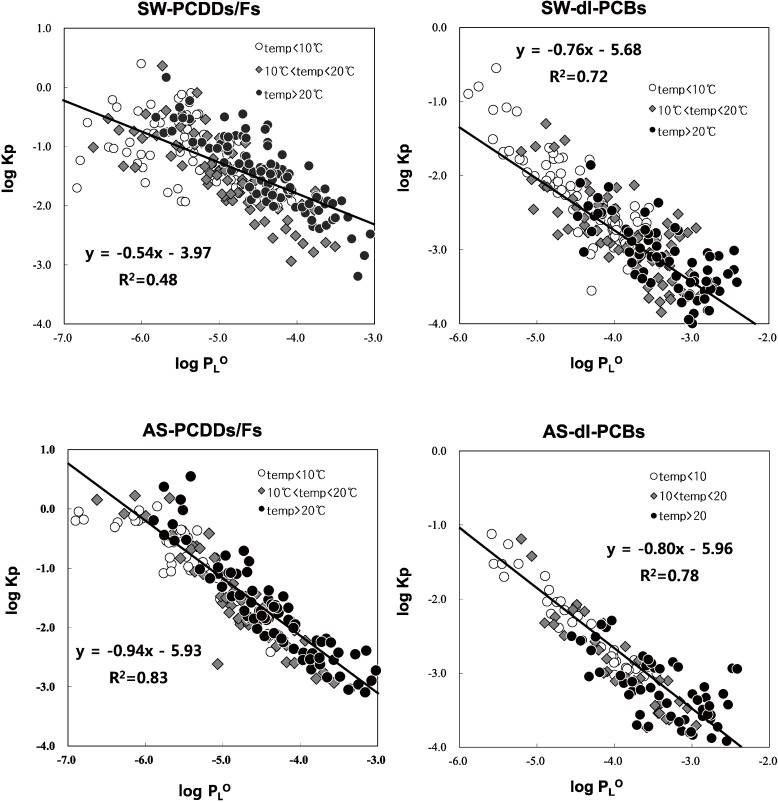
Slope (m), intercept (b), correlation coefficient (R^2^), and plot of log Kp *vs.* log P_L_° for PCDDs/Fs and dl-PCBs at two sampling sites.

Figure 2 and Table 3 illustrate the log plot of K_p_ *vs.* P_L_° for PCDDs/Fs (2,3,7,8 substituted congeners) and dl-PCBs in Suwon and Ansan air. For PCDDs/Fs and dl-PCBs, this plot yield slopes of −0.53 and −0.76 for Suwon (urban-residential area) and −0.94 and −0.80 for Ansan (industrial area), respectively. PCDDs/Fs and dl-PCBs adsorbed onto particles from first-combustion emission sources were emitted into the atmosphere, resulting in the slope for the Ansan data (where the majority of emission sources are located) being steeper than that for the Suwon data. At high temperatures (over 20 °C), the slope is close to −1, indicating equilibrium absorptive partitioning with good correlation. However, at low temperatures (below 10 °C), the slope (m) for PCDDs/Fs is less steep in the winter season, resulting in a reduction in the correlation coefficient (R^2^) between log K_p_ and log P_L_°. This phenomenon is assumed to be caused by the non-equilibrium gas-particle partitioning that occurs under cold temperature conditions, likely owing to enhanced emissions of PCDD/Fs to the air mass at cold temperatures due to seasonally dependent combustion-related sources [51]. Under such conditions, the log (F/TSP)/A would be lower than expected at equilibrium, inducing a shallow slope (m) and high intercept (b). In contrast to PCDDs/Fs, the slope for PCBs in summer is relatively shallow compared to that in winter, and gas-phase PCB concentration should increase with increasing ambient air temperature [42] owing to desorption from the particle phase, resulting in a low log (F/TSP)/A value and shallow slope. Thus, the difference in K_p_ values for PCB and PCDDs/F partitioning likely reflect the physicochemical characteristics of the different compound categories.

**Table 3:** Detailed fitting parameters for the log Kp *vs.* log P_L_° plots for PCDDs/Fs and dl-PCBs at the two sampling sites.

Compound	Sampling Site	Slop (m)	Intercept (b)	R^2^	Level of Significance	Sampling Site	Slop (m)	Intercept (b)	R^2^	Level of Significance
PCDDs/Fs	SW (all temp.)	−0.54	−3.97	0.48	<0.001	AS (all temp.)	−0.94	−5.93	0.83	<0.001
	SW (temp. <10 °C)	−0.32	−2.85	0.20	<0.001	AS (temp. < 10 °C)	−0.82	−5.38	0.76	<0.001
	SW (10 °C < temp. < 20 °C)	−0.75	−5.17	0.62	<0.001	AS (10 °C < temp. < 20 °C)	−1.16	−7.13	0.85	<0.001
	SW (temp. > 20 °C)	−0.86	−5.24	0.75	<0.001	AS (temp. > 20 °C)	−1.09	−6.15	0.84	<0.001
dl-PCBs	SW (all temp.)	−0.76	−5.68	0.72	<0.001	AS (all temp.)	−0.80	−5.96	0.78	<0.001
	SW (temp. < 10 °C)	−0.84	−5.97	0.74	<0.001	AS (temp. < 10 °C)	−0.88	−6.25	0.93	<0.001
	SW (10 °C < temp. < 20 °C)	−0.70	−5.50	0.56	<0.001	AS (10 °C < temp. < 20 °C)	−0.99	−6.79	0.83	<0.001
	SW (temp. > 20 °C)	−0.66	−5.34	0.51	<0.001	AS (temp. > 20 °C)	−0.56	−5.17	0.43	<0.001

### 3.4. PCDDs/Fs and PCBs Profiles in Emission Sources and Ambient Air

To evaluate the relative contributions from emission sources, the fraction of the congeners in an emission source is often used, given as Equation (2):

The congener fraction in combined emission sources (%) = ∑(C_congener_·i × f_CR_·i)/∑ (C _total_·i × f_CR_·i) (2)

where C_congeners_ (μg·m^−3^) is the average congener concentration in flue gas emitted from facilities, f_CR_ (%) is the contribution ratio in combined profile from all sources for PCDDs/F and dl-PCBs, and C_total_ is the total PCDDs/Fs in flue gas emitted from various facilities.

**Figure 3:**
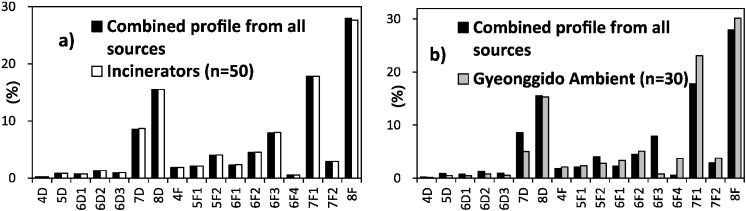
Comparison of the PCDD/F congeners’ pattern, (**a**) combined profile from all sources (black) *vs.* incinerator profile (white); (**b**) combined profile from all sources (black) *vs.* ambient air (grey).

The concentrations of PCDDs/Fs and dl-PCBs (C_total_) in various emission sources in Korea were compiled from previous studies [52,53], and the emission contribution ratio is based on the inventory of source dioxin in Korea [54]. Figure 3 illustrates a comparison of the PCDD/F profile with incinerator emissions and with the profile of ambient air data collected in Gyenggi-do Province. The calculated congener profile of all sources combined is particularly similar to the profile for flue gas of average incinerator emissions, including municipal solid waste incinerators and hazardous waste incinerators, as we expected. Moreover, the PCDD/F congener profile of all sources combined is in good agreement with the ambient air profile measured in the present study. OCDF, OCDD, 1,2,3,4,6,7,8-HpCDF, and 1,2,3,4,6,7,8-HpCDF were found in relatively high portions in both the flue gas of emission sources and ambient air. This implies that combustion processes and incinerators were the primary sources for the PCDD/F congener profile in the sampled air. Furthermore, the secondary sources of PCDDs/Fs (such as volatilization in soil and sediment) were insignificant, because the PCDDs/Fs were well “trapped” in the interiors of particles and strongly adsorbed to soil.

**Figure 4:**
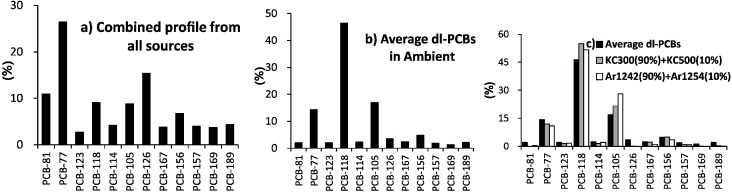
Distribution of dl-PCB congeners’ pattern, (**a**) combined profile from all sources; (**b**) Gyeonggi-do ambient air; (**c**) comparison of the dl-PCB congeners pattern in ambient air with a mixture of Kanechlor (KC-300 and KC-500) and Aroclor (Ar-1242, Ar-1254).

Figure 4 compares the dl-PCBs in combined emission sources of various facilities with those obtained for ambient air in the present study. Using Equation (2), we calculated the relative fraction of dl-PCBs in emissions. The non-ortho dl-PCBs in flue gas were dominated by compounds such as PCB77, PCB-126, and PCB-81, whereas the mono-ortho PCB-118 was the dominant dl-PCB in ambient air. Previous studies have indicated that PCBs are introduced to air primarily from the disposal of electrical equipment or by re-emission from soil, with emissions varying as a function of air temperature [55,56].

Long-term PCBs in atmospheric environments tend to reflect trends in the production and use of PCBs [57]. The congener profiles of the dl-PCBs considered in the present study were compared to two existing PCB congener fractions of Kanechlor (KC) [58] and Aroclor (Ar) [59]. We initially compared the measured dl-PCB congener profiles with those in KC commercial products such as KC-300, KC-400, and KC-500, in mixing proportions determined by a linear regression method. A mixture the KC-300 (90%) and KC-500 (10%) was found to be similar to the dl-PCB profile of ambient air. The mixture of Ar-1242 (90%) and Ar-1254 (10%) also exhibited similarity to the dl-PCB profile of ambient air, because the chlorine contents of Ar-1242, Ar-1248, and Ar-1254 closely match those of KC-300, KC-400, and KC-500 [60,61].

The congener pattern of the PCBs in ambient air excluding PCB-11 agreed well with a 90:10 mixture of KC-300 (Ar-1242) and KC-500 (Ar-1254). The correlation coefficients (R^2^) of the congener profiles between ambient air and two products (KC and Ar) were 0.85 and 0.84, respectively, and these relationships were statistically significant (*p* < 0.0001). The Monsanto company produced the Aroclor formulations accounting for more than 50% of the reported historical production of Ar-1254 (1952 to 1955), Ar-1242 (1955 to 1971), and Ar-1016 (1971 to 1977) in the USA [62]. We were able to reproduce the PCB profile of the ambient air using simple mixtures of Ar-1242 (KC-300) and 1254 (KC-500), the production and usage of which were halted long ago. These results indicate that historically accumulated burdens in soils and the ground could be a major source of PCBs in ambient air, particularly during hot periods.

## 4. Conclusions

PCDD/F and dl-PCB concentrations for Ansan (an industrial area) were higher than those for Suwon (an urban area). However, total PCB and ICES-PCB concentrations exhibited similar values in both areas. In particular, PCB-11 made up more than 15% of Σ PCBs and demonstrated its possible application as an indicator to infer total PCB levels, calculated to be 3–4 times the PCB-11 level in these sites in Korea. The slopes (m) for a log K_P_ *vs.* log P_L_° plot for industrial air for PCDDs/Fs and dl-PCBs were steeper than those for urban air owing to the presence of local sources in industrial areas. At air temperatures above 20 °C, the regression log K_P_ *vs.* log P_L_° plot for PCDDs/Fs demonstrated good correlations with steep slope. This indicates additional emissions from combustion-related sources at cold temperatures, where PCDDs/Fs diverged from the regression line of particle-gas partitioning. Using our measured PCDD/F and PCB congener profiles and existing emission factors (contribution ratios) from available sources in Korea, we were able to estimate their relative contributions from diverse emission sources. PCDDs/Fs and PCBs were affected primarily by direct emission sources and secondary re-emissions by soil volatilization, respectively. We found that incinerators were the largest emission source for atmospheric PCDDs/Fs, whereas re-evaporation from pre-existing environmental loads was the primary source for atmospheric PCBs.
